# Effect of Third-Generation Beta Blockers on Weight Loss in a Population of Overweight-Obese Subjects in a Controlled Dietary Regimen

**DOI:** 10.1155/2021/5767306

**Published:** 2021-09-23

**Authors:** Maria Alessandra Gammone, Konstantinos Efthymakis, Nicolantonio D'Orazio

**Affiliations:** ^1^Human and Clinical Nutrition Unit, Department of Medical Oral and Biotechnological Sciences, G. D'Annunzio University, Via Dei Vestini 31, Chieti 66013, Italy; ^2^Department of Medicine and Ageing Sciences and Center for Excellence on Ageing and Translational Medicine (CeSI-MeT), G. D'Annunzio University and Foundation, Via Luigi Polacchi 11, Chieti 66013, Italy

## Abstract

**Background:**

Overweight and obesity often develop in individuals with genetic susceptibility and concomitant risk factors; however, medications can represent precipitating factors in some cases: evidence suggests that some antihypertensive drugs can adversely affect energy homeostasis and metabolism.

**Aim:**

The primary aim of this study was to investigate whether long-term therapy with a beta blocker impairs weight loss during a period of appropriate personalized hypocaloric diet and standardized physical activity in overweight and obese hypertensive patients in monotherapy and without comorbidities, compared to other antihypertensive drugs and to a control group not taking antihypertensive therapy. *Subjects and Methods*. We enrolled overweight and obese patients taking antihypertensive drugs; subjects were divided into 3 groups: those taking traditional beta blockers (bB group), those taking third-generation beta blockers (bB-3 group), and those taking other antihypertensive drugs (non-bB group). We also enrolled subjects receiving neither antihypertensive therapy nor other chronic medication in the prior 12 months as controls. All subjects underwent personalized hypocaloric diets for a period of 24 months with monthly follow-up. Anthropometric parameters were measured at enrollment and then monthly after diet prescription. Glucose and lipid values were assessed at baseline and at 12 and 24 months during dietary regimen.

**Results:**

We enrolled a total of 120 overweight and obese patients aged 50.30 ± 1.13 years (mean ± standard deviation) with a mean BMI of 31.79 ± 0.65 kg/m^2^; 90 were taking antihypertensive drugs (no comorbidity and no polytherapy), while 30 subjects receiving neither antihypertensive therapy nor other chronic medication in the prior 12 months were considered as controls. After 6 months, the percent total weight loss (TWL%) was lower in the bB group (3.62 ± 1.96 versus 5.27 ± 1.76 in the bB-3 group, versus 5.15 ± 1.30 in the non-bB group, and versus 4.70 ± 0.87 in the control group), as well as their BMI. After 24 months, we kept finding the worst result in the bB group (TWL% = 9.22 ± 2.19 versus 12.79 ± 1.72 in the non-bB group and 12.28 ± 1.97 in the control group) with the best trend in the bB-3 group (TWL% = 16.19 ± 2.67).

## 1. Introduction

The global tendency towards lower physical activity and increased dietary intake of fats, sugars, and calories is leading to a growing prevalence of overweight, obesity, and lifestyle-related metabolic diseases, such as diabetes, hypertension, dyslipidemia, and metabolic syndrome. Obesity is a worldwide epidemic and is associated with significant morbidity and mortality [1, 2]. The causes of obesity are multifactorial, with dietetic habits, lifestyle, and genetic background being the most widely researched. Overweight and obesity often develop in individuals with genetic susceptibility; however, some drugs are among the recognized precipitating factors, even though the possibility that medication use may contribute to obesity has received little attention.

The sympathetic nervous system plays a pivotal role in the maintenance of body weight, stimulating energy expenditure, and fat utilization [3]. It has been hypothesized that a causative relationship between a low sympathetic drive and the development of obesity may exist [4–7]. The importance of the sympathetic nervous system in the regulation of energy homeostasis has been highlighted in animal studies, showing that obesity can certainly develop from a reduction in energy expenditure induced by ablation of adrenergic receptors [8]. In humans, beta blockers acutely blunt energy expenditure, substrate utilization, and aerobic exercise capacity [9, 10]. However, chronic adrenergic inhibition results in beta adrenoceptor upregulation in animals, indicating possible loss of efficacy [11]. The extent to which chronic beta blocker therapy impairs energy expenditure and physical activity, in otherwise healthy individuals, has not been investigated. Beta blockers are commonly prescribed to patients for hypertension, which frequently occurs with many other comorbidities. The level of physical activity among patients with chronic comorbidities, who as a group tend to be sedentary, may be further restricted. Thus, there is a strong possibility that medications, which chronically blunt sympathetic nervous system activity, may favor weight gain and predispose to overweight and obesity. In this respect, blood pressure management in hypertensive patients with metabolic abnormalities is challenging, since many of the antihypertensive drugs adversely affect metabolism. Medications that interfere with weight loss are of obvious concern in the management of obese patients. Such medications can often be avoided or substituted by alternatives. In previous studies [9–11], beta blockers have been associated with long-term weight gain, but little is known about the effect of beta blockers on weight loss in response to a precise hypocaloric regimen. The obesity-promoting impact of beta blockers is not currently well understood. Resting energy expenditure and thermogenesis induced by external factors [12], such as meals and cold exposure, but also stress and anxiety, have a facultative component mediated by the sympathetic-adrenal system through catecholamines working on beta adrenoceptors. Treatment with beta blockers reduces the facultative thermogenesis by 50–100 kcal/day, which corresponds to the weight gain of 2–5 kg/year reported in clinical trials [12]. It can also result in insulin resistance, which may aggravate existing diabetes and elicit diabetes in predisposed patients. Overweight and obesity are frequently complicated with hypertension, which is often treated by beta blockers. Obesity is associated with a defective sympathetic activity, and treatment with beta blockers may further reduce facultative thermogenesis and promote weight gain. The consequence may be aggravation of hypertension, insulin resistance, and other metabolic disturbances. *β*-Adrenoceptor antagonists have historically been considered an effective and safe option for first-line treatment of hypertension, but very recently, it has been proposed that *β*-blockers should no longer be considered suitable for first-line therapy in the patient with uncomplicated hypertension because of unfavorable metabolic effects [12].

However, some properties could consistently differentiate third-generation beta blockers, such as nebivolol and carvedilol, from classical nonvasodilating beta blockers, such as atenolol, metoprolol, or bisoprolol. Besides effective control of blood pressure in patients with hypertension, third-generation vasodilating beta blockers may offer additional benefits for central hemodynamics and neutral or beneficial effects on metabolism [13].

## 2. Study Design, Subjects, and Methods

The primary aim of this study was to investigate whether long-term therapy with a beta blocker impairs weight loss during a period of appropriate personalized hypocaloric diet and standardized physical activity in overweight and obese hypertensive patients in monotherapy and without comorbidities, compared to other antihypertensive drugs and to a control group not assuming any medication. Patients under diuretic treatment were not considered for this study. The secondary aim was to evaluate any differences in weight loss among beta blocker users, specifically regarding third-generation beta blockers, such as nebivolol and carvedilol. The present study will also summarize the available clinical evidence regarding the metabolic effects of beta blockers in hypertensive patients, with an emphasis on third-generation beta blockers.

We compared total weight loss (TWL) between beta blocker users and subjects who were treated with other antihypertensive drugs. Two aspects of metabolic rate, BMR and physical activity, were assessed. The indication for beta blocker therapy was hypertension in every case. Participants were enrolled consecutively and were in otherwise good health, all leading independent lives and not functionally restricted. Individuals were excluded if they had any significant organ dysfunction, were receiving any other medications (that could confer divergent effects on substrate oxidation and body composition [14], potentially altering metabolic rate and interfering with our results), and had congestive heart failure, hepatic or renal disease, or a malignancy. Basal metabolic rate (BMR) was assessed by bioimpedentiometric analysis after an overnight fast. Energy requirements were calculated by the Mifflin equation, which is the best predicting equation for estimating energy expenditure in people with BMI < 40 kg/m^2^ [15].

Patient characteristics, including age, sex, past medical history, and current medications, were recorded. Duration of continuous antihypertensive treatment was determined by reviewing medical records of individual users dating back more than 2 years.

Each patient underwent a preliminary medical visit, in order to collect their personal and family anamnesis, as well as a nutritional 24-hour and 7-day recall, in order to quantify their caloric intake and dietary habits.

Subjects also underwent a Stanford 7-day recall in order to assess habitual physical activity and to quantify their additional energy expenditure. The 7-day activity recall questionnaire is designed specifically for mature subjects, assessing participation in household chores and leisure activities. Participants were asked to recall morning, afternoon, and evening activities for a week. Cues were used to prompt classification of activity into their respective intensities. Direct questions considered time (minutes per day) spent at light (1.2–2 MET), moderate (4 MET), hard (6 MET), or very hard (10 MET) physical activities and time (hours per day) spent asleep (1 MET) [16]. The physical activity level resulted to be at baseline (1.2 MET) for each one because of their sedentary lifestyle. Baseline activity refers to the light-intensity activities of daily life, such as standing, walking slowly, and lifting lightweight objects [17]. Energy expenditure of modern humans is generally low because of a general tendency to adopt sedentary lifestyles, with motorized transport, domestic appliances, and mechanized equipment displacing physical exercise and manual work. People reporting only baseline activity are considered to be inactive: any occasional or short episodes of moderate/high intensity activity, such as climbing stairs or walking in a fast pace, are not considered long or frequent enough to count. Personalized dietetic therapies were prescribed according to their calculated basal metabolic rates and estimated level of physical activity.

Anthropometric parameters (weight, height, and waist and hip circumferences) were measured. Body weight and height were measured using the same equipment for all subjects. Waist circumference was measured using a meter tape placed on a horizontal plane at the level of the iliac crest as seen from the anterior view. All measurements were performed by the same clinician.

Glucose and lipid values were assessed before dietetic therapy and after 12 and 24 months, always remaining in the normality range. Subjects were divided into 4 groups: those taking traditional beta blockers (bB group), those taking third-generation beta blockers (bB-3 group), those taking other antihypertensive drugs, represented by sartans, angiotensin-converting enzyme inhibitors, and calcium channel blockers (non-bB group), and patients with normal blood pressure values not taking any antihypertension medication (controls). All groups underwent hypocaloric diets for a period of 24 months with regular monthly follow-up. Differences in proportions between groups were assessed by the *χ*^2^ test. Differences in continuous variables were assessed by analysis of variance (ANOVA), applying Welch's correction where needed. Post hoc analysis was performed by the Bonferroni or Dunnett's test as appropriate. Statistical significance was considered at the 95% level (*p* < 0.05). Statistical analysis was performed with the use of the IBM SPSS Statistics for Windows Software Suite, version 20.0, IBM Corporation, Armonk, NY.

## 3. Results

**Figure 1:**
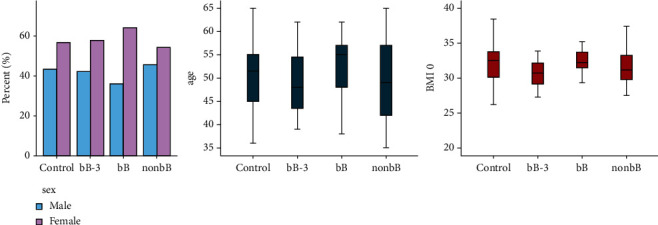
Sex distribution, age distribution, and baseline BMI (BMI 0) in the study population: the enrolled patients, aged 50.30 ± 1.13 years, had a mean BMI of 31.79 ± 0.65 kg/m2; 90 subjects (F : *M* = 1.36) were taking antihypertensive drugs; 30 subjects (M : *F* = 1.31), not receiving any antihypertensive therapy in the past 12 months, were enrolled as controls.

We enrolled consecutively 120 overweight and obese patients aged 50.30 ± 1.13 years (mean ± standard deviation) with a mean BMI of 31.79 ± 0.65 kg/m^2^. Of those, 90 subjects (52 females and 38 males, F : M = 1.36) were taking antihypertensive drugs, while 30 overweight/obese patients (17 females and 13 males, M : F = 1.31), not receiving either antihypertensive therapy or other chronic medication in the past 12 months, were enrolled as controls (Figure 1). Patients using diuretics or multiple antihypertensive drugs or presenting comorbidities were not recruited as per the inclusion and exclusion criteria of our study.

Female subjects represented 64% of the Bb group, 57.9% of the bB-3 group, 54.3% of the non-bB group, and 56.7% of the control, while males represented, respectively, 36%, 42.1%, 45.7%, and 43.3% of these groups. Differences were not significant for the *χ*^2^ test (*p* > 0.05). Mean age differences were also not significant (*p* > 0.05), with a mean age of 50.57 years (± 7.38) among the controls, 49.16 years (± 7.33) among bB-3, 52.04 years (± 6.71) among bB, and 49.46 years (± 8.46) among non-bB group.

The BMI also showed no significant differences at baseline (*p* > 0.05): its mean value was 32.12 kg/m^2^ (± 3.02) for the control group, 30.72 kg/m^2^ (± 1.98) for bB-3, 32.43 kg/m^2^ (± 1.67) for bB, and 31.88 kg/m^2^ (± 2.72) for non-bB. The differences in waist circumferences (WC) were also not significant at baseline (*p* > 0.05), with a mean value of 102.20 cm (± 12.30) in controls, 101.26 cm (± 8.61) in bB-3, 105.08 cm (± 6.29) in bB, and 102.91 cm (± 10.23) in non-bB.

We found a significant difference in weight loss in the different groups during our follow-up.

**Figure 2:**
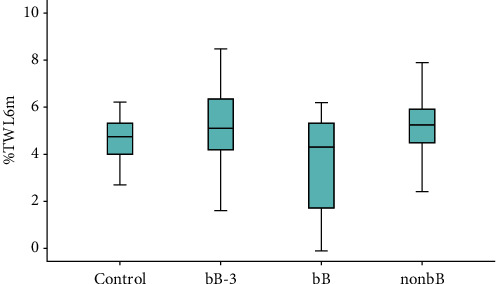
Percentage of total weight loss (TWL%) after 6 months in the 4 groups: after 6 months, we found a lower TWL% in bB (3.62 ± 1.96 versus 5.27 ± 1.76 in the bB-3 group, versus 5.15 ± 1.30 in the non-bB group, and versus 4.69 ± 0.87 in the control group).

After 6 months (Figure 2), the total weight loss was lower in bB (TWL% mean value = 3.62 ± 1.96 versus 5.27 ± 1.76 in the bB-3 group, versus 5.15 ± 1.30 in the non-bB group, and versus 4.69 ± 0.87 in the control group). BMI displayed a similar difference: 31.27 kg/m^2^ ± 1.82 in the bB group, 29.10 ± 1.85 in the bB-3 group, 30.25 ± 2.67 in the non-bB group, and 30.62 ± 2.90 in the control group.

**Figure 3:**
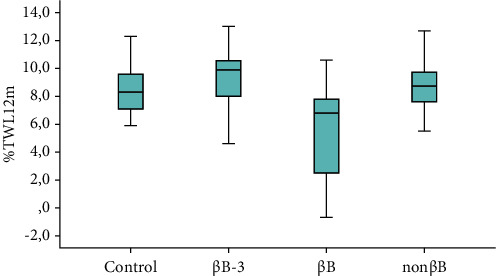
Percentage of TWL after 12 months in the 4 groups: the mean TWL% value of the bB group after 12 months resulted to be 5.69 ± 3.33, versus 8.83 ± 1.70 of the non-bB group and 8.54 ± 1.63 of controls.

After 12 months, bB patients kept showing the worse results with a mean BMI value of 30.60 kg/m^2^ ± 1.94, versus 29.07 ± 2.61 of non-bB and 29.39 ± 2.88 of controls. Similarly, the mean TWL% value of the bB group after 12 months resulted to be 5.69 ± 3.33, versus 8.83 ± 1.70 of the non-bB group and 8.54 ± 1.63 of controls (Figure 3). Meanwhile, bB-3 patients were standing out positively, with the lowest mean BMI (27.81 kg/m^2^ ± 1.71) and the highest mean TLW% (9.45 ± 2.17).

After 24 months, we kept finding the worst result from the bB group (BMI mean value = 29.44 kg/m^2^ ± 1.64 versus 27.82 ± 2.56 in the non-bB group and 28.19 ± 2.74 in the control group; TWL% = 9.22 ± 2.19 versus 12.79 ± 1.72 in the non-bB group and 12.28 ± 1.97 in the control group), with the best trend in the bB-3 group (BMI = 25.72 kg/m^2^ ± 1.32; TWL% = 16.19 ± 2.69).

Waist circumference showed a similar trend: after 6 months, its mean value was 100.75 cm ± 6.84 in bB patients, versus 96.43 cm ± 8.41 in bB-3, versus 98.71 cm ± 10.37 in non-bB, and versus 96.43 cm ± 10.37 in controls. After 24 months, its mean value was always higher in bB patients (96.13 cm ± 5.31), compared to non-bB (90.24 cm ± 7.99), bB-3 (90.29 cm ± 5.31), and controls (87.57 cm ± 7.74), whose mean value resulted to be the most favorable in this case.

## 4. Discussion

We hypothesize that chronic traditional *β*-blockade favors overweight by blunting energy expenditure.

The main limitation of this study consists in its nonrandomization: a randomized trial could not be carried out because the specific aim was to evaluate the effects of different treatments. On the other side, without random allocation, treatment effects are likely to be affected by systematic bias or increased uncertainty: this leaves open the possibility that those who are on a particular beta blocker may have different features. However, other previous investigations displayed an association between chronic traditional beta blocker therapy and overweight. In these studies, in spite of an unaltered energy intake, the reduction in habitual activity accounted for significant weight gain in the long term. It is likely that beta-blocker-induced reduction in exercise capacity, even in daily activities, diminishing overall energy expenditure and lipid mobilisation, may accentuate weight gain in these patients [18, 19]. The dynamics of beta-blocker-related weight gain has been examined by Sharma et al. [20]. Based on weight regression analysis, they concluded that weight gain occurred predominantly in the first year of beta blocker use [21–24]. In particular, beta blocker users failed to lose weight, remaining 6% heavier than nontreated participants [25–29]. The reason for the maintenance of a higher weight during traditional beta blocker therapy is unclear but may be secondary to metabolic maladaptations and establishment of a new weight “set-point” [20, 30], so that chronic traditional beta blocker users are more obese because they exhibit reduced levels of diet-induced thermogenesis, fat utilization, and physical activity, changes that lead to the development of obesity.

The class of beta blockers can be dichotomized based on their ability to vasodilate [31, 32] versus having limited or no vasodilatory properties. The vasodilators include carvedilol, labetalol, and nebivolol; the nonvasodilating beta blockers include the traditionally used metoprolol, atenolol, propranolol, and bisoprolol. The mechanism of action for arteriolar dilatation is different among the vasodilating beta blockers: arteriolar dilatation occurs through either direct blockade of the alpha-1 receptors (labetalol and carvedilol) or endothelial-derived NO via stimulation of endothelial-derived nitric oxide synthase (eNOS) with nebivolol [33].

Third-generation beta blockers, such as carvedilol and nebivolol (bB-3 group), exert neutral (after 6 and 12 months) and even beneficial effects (after 24 months) in hypertensive overweight patients following hypocaloric dietotherapy, probably due to their nitric oxide-mediated vasodilatory and antioxidative properties. In particular, recent clinical studies about nebivolol, a third-generation highly selective *β*1-blocker with additional endothelial NO-mediated vasodilating activity, confirm previous findings that this drug differs from other beta blockers. The combined mechanisms of *β*-adrenoceptor antagonism and NO-mediated vasodilation may potentiate the blood pressure-lowering effect of this agent and confer a broader favorable metabolic profile, which may be clinically relevant for hypertensive patients. Recent studies particularly focused on nebivolol, a highly cardioselective vasodilator represented by a racemic mixture of D- and L-enantiomers, which may benefit patients with cardiometabolic risk [34, 35]. Its vasodilatory properties are due to its important role of endothelin-derived nitric oxide and its activation of beta 3-adenoreceptor [33, 35, 36]. Nebivolol has enhancing effects via several methods including the suppression of asymmetric dimethyl arginine (ADMA), an endothelial nitric oxide synthase (eNOS) inhibitor [37, 38]. In addition, nebivolol's vasodilating and antioxidant properties, mediated by NO, demonstrated benefits on other components of cardiometabolic risk, such as insulin sensitivity and lipid profile. One study comparing nebivolol with nonvasodilatory beta blockers evidenced a worsening of glycosylated hemoglobin (HbA1C) after a 6-month intervention with the latter group [39–41]. Another study of approximately 5000 patients with hypertension and type 2 diabetes demonstrated reductions in mean plasma glucose in those treated with nebivolol as monotherapy (21.4%), although 48.4% were also being treated with an ACE inhibitor or angiotensin receptor blocker (ARB) [38–41]. Recent animal studies also evaluated the effect of nebivolol: Zucker obese rats were used to investigate regulation of the obesity promoter miR-208a. Nebivolol-induced suppression of cardiac miR-208a and increase in MED13 were correlated with attenuated weight gain despite leptin resistance; consequently, resistance to obesity was observed in rodents treated with nebivolol [42]. In one small randomized intervention study with metoprolol versus nebivolol, despite similar BP reduction in both groups, metoprolol decreased insulin sensitivity compared with nebivolol; in addition, ADMA, a reflection of oxidative stress and augmentation index, increased in a dose-dependent manner by metoprolol but not with nebivolol [13]. In contrast, carvedilol attenuated its increase in the failed ventricle. Improved cardiac output seen with carvedilol allows for improvement in insulin sensitivity with a beneficial effect on cardiometabolic risk [43, 44]. In fact, in the Carvedilol Or Metoprolol European Trial (COMET), a study of over 3000 subjects, carvedilol was shown to reduce risk for the development of new diabetes by 22% [40, 41, 43, 45]. Furthermore, in the Glycemic Effects in Diabetes Mellitus (GEMINI) trial comparing carvedilol and metoprolol on glycemic control in 1235 participants with hypertension and type 2 DM (HbA1c: 6.5–8.5%) on top of renin angiotensin RAS blockers, 40% fewer patients progressed to microalbuminuria in the carvedilol arm than in the metoprolol arm in hypertensive patients [46].

Carvedilol showed a neutral or favorable effect on levels of both triglycerides and high-density lipoprotein cholesterol [47]. Unlike nebivolol, the NO-mediated effects of carvedilol are less well described. Carvedilol is lipophilic and is concentrated in lipid membranes; this facilitates fatty acid peroxidation [43, 48, 49]. Carvedilol has also shown benefit in the COPERNICUS study and is likely to be used in the setting of heart failure. Nebivolol acts as a zwitterionic acid, donating electrons to oxygen free radicals thereby reducing oxidative stress [50, 51]. Additionally, it may have anti-inflammatory pleiotropic benefits, which may be protective for atherosclerotic disease [42, 52]. Ayers et al. similarly demonstrated a significant relative increase in oxidative stress for metoprolol compared with nebivolol, as well as differential effects of nebivolol and metoprolol on insulin sensitivity and plasminogen activator inhibitor in the metabolic syndrome measured by F2-isoprostanes, after just a 12-week period of randomization [37]. Thus, the antioxidant properties of nebivolol and carvedilol and their neutral or even favorable effects on both carbohydrate and lipid metabolism are well documented. Thus, third-generation beta blockers could be a favorable therapeutic option for the treatment of hypertension in overweight and obese patients, especially those with impaired glucose and lipid metabolism. Awareness of these facts and highly individualized dietetic therapy, as well as drugs prescription, seems to be the way forward. Concerning blood pressure control, it should be based on lifestyle changes, diet, adequate daily water intake, and physical exercise, which allow for weight reduction and improve muscular blood flow. If any antihypertensive drugs are strictly necessary, angiotensin-converting enzyme inhibitors, angiotensin II-AT1 receptor blockers, or even calcium channel blockers are preferable over classical beta blockers, if no compelling indications are present for their use.

## Data Availability

The data are available from the corresponding author upon request.
